# Supplementary material for: A behavioral and genetic study of multiple paternity in a polygamous marine invertebrate, Octopus oliveri
Source: PeerJ. 2019 Jun 7;7:e6927. doi: 10.7717/peerj.6927 (PMC6557246; doi:10.7717/peerj.6927)
Supplement: Supplemental Information 4 — Additional figures, analyses and a complete list of microsatellite loci and primers discovered for Octopus oliveri to support the presentation of data in the primary manuscript. [file peerj-07-6927-s004.docx]

**Supplementary material**


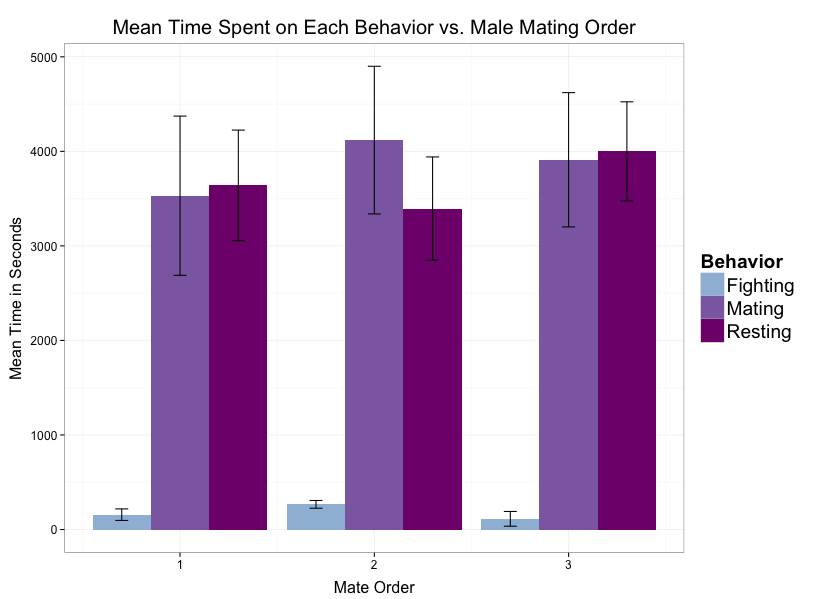


S1: Mean time (in seconds) spent on fighting, mating, or resting for all male *Octopus oliveri* from each category of male order (first, second, third). Error bars represent standard error.


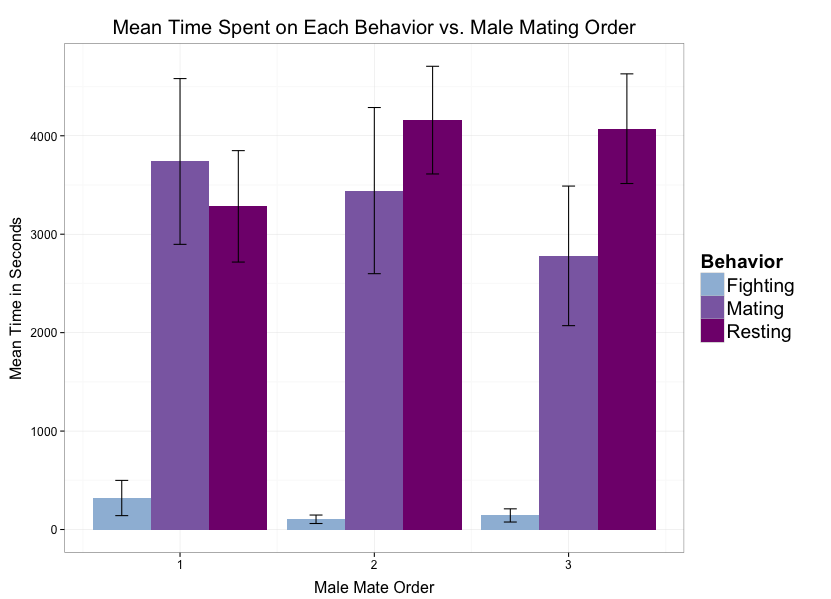


S2. Mean time (in seconds) spent on fighting, mating, or resting in individual male *Octopus oliveri* between given mate order. Error bars represent standard error.


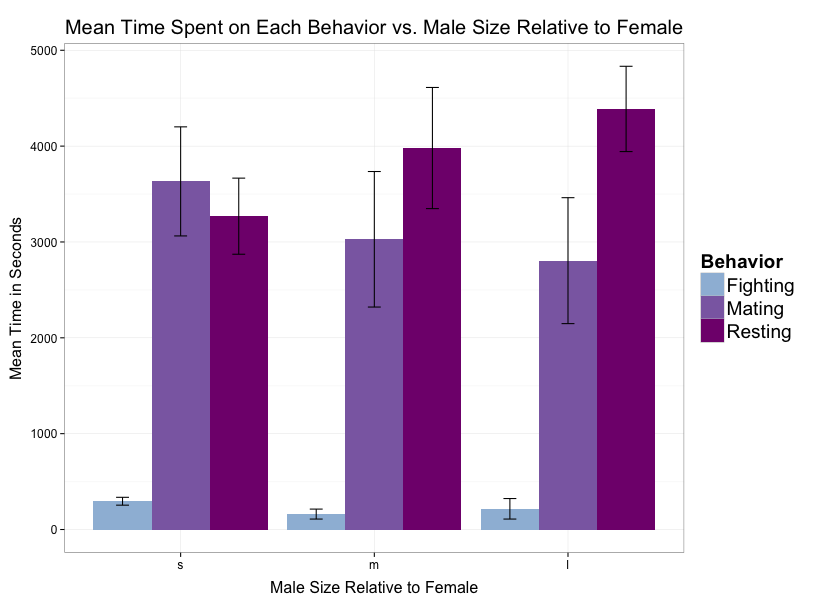


S3. Mean time (in seconds) spent on fighting, mating, or resting when comparing male size relative to female. Size s: small, m: medium, l: large. Error bars represent standard error.


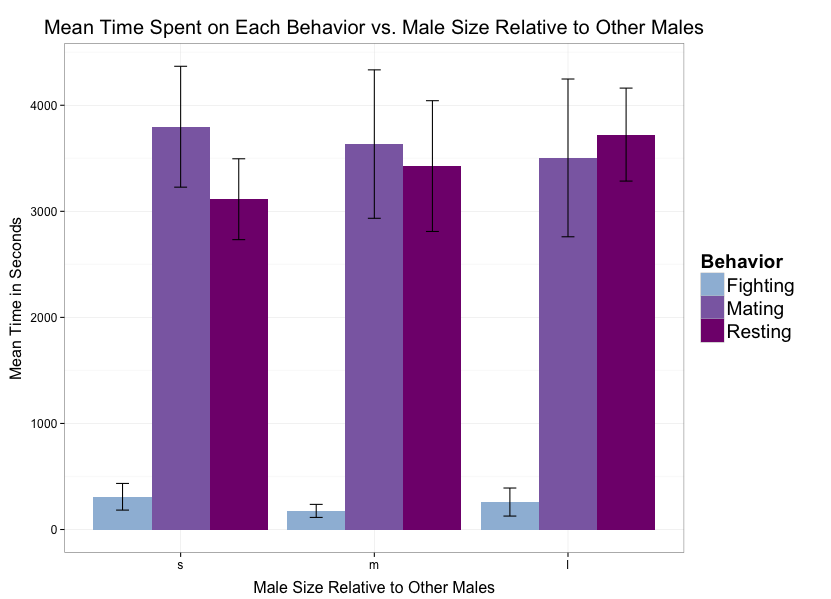


S4. Mean time (in seconds) spent on fighting, mating, or resting when comparing male size relative to other male *Octopus oliveri* in the population. Size s: small, m: medium, l: large. Error bars represent standard error.


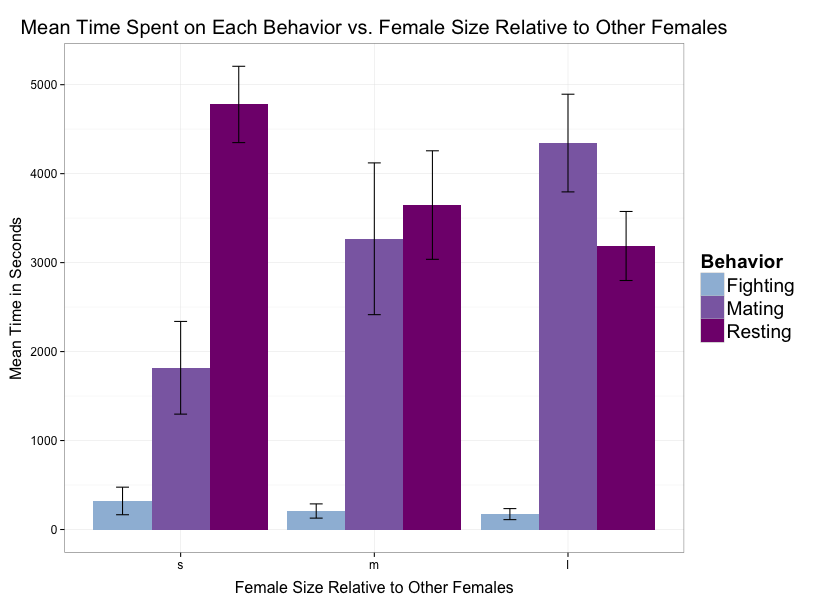


S5. Mean time (in seconds) spent on fighting, mating, or resting when comparing female size relative to other female *Octopus oliveri* in the population. Size s: small, m: medium, l: large. Error bars represent standard error.

S6. 48 species-specific microsatellite loci isolated for *Octopus oliveri*. Of the initial 48 loci identified (Fernandez-Silva et al. 2013) the first 5 to work (denoted in *bold below) were used in this study because they amplified consistently and provided the exclusion power needed for this work (see S7).

| **Locus** | **size** | **Motif** | **Primer Sequence (5'-3')** |
| --- | --- | --- | --- |
| Octoli_001 | 4 | ACAT | F: GCATTAAGTCATCAACATGC |
|  |  |  | R: ATGCCAGCATGGTACTTC |
| Octoli_002 | 4 | ATCT | F: CACAGATACATCCGCACACG |
|  |  |  | R: TTGAGAGACGCCGACCAC |
| ***Octoli_003** | 4 | TAGA | F: GCACGTTGTACGCGATTC |
|  |  |  | R: ATATGCATGAAGACGCAACTC |
| Octoli_004 | 4 | ATAC | F: TGCTTCTCAACCACATGTATGC |
|  |  |  | R: ACGCACACACTTGATGTAGAGTTC |
| Octoli_005 | 4 | ATCT | F: ACCGTCATCTATCAATCCATC |
|  |  |  | R: TGCTAGATATGTTCATATTGAGAACT |
| Octoli_006 | 4 | ATAG | F: TTATGCGCTTAATTCGGTAT |
|  |  |  | R: CTTCTCATTCTCCAACATTGA |
| ***Octoli_007** | 4 | TATG | F: CGCAGACGAGGAATCAATAG |
|  |  |  | R: GGAGAACAGACACAAGAACACAG |
| Octoli_008 | 4 | ATCT | F: CATGAATGCAGTATTAGTATGA |
|  |  |  | R: AACTTAGTCTCTCCACTCTGAT |
| Octoli_009 | 4 | AGAT | F: CGATCTGAGCCAGCACTTC |
|  |  |  | R: TCCACACACACGTATATTATATGGAC |
| Octoli_010 | 4 | CATA | F: CGCCAGTGATTGAACTTATGAC |
|  |  |  | R: ATCAGGAAGAGGTGGTGCAG |
| Octoli_011 | 4 | CATA | F: ACCATCCATCCATCCATCC |
|  |  |  | R: GTGTGAGCTCGTGAGTGAGG |
| Octoli_012 | 4 | TATG | F: GTGTGAGTGCATATGTGTG |
|  |  |  | R: CACTAGCGATACGATACATAA |
| Octoli_013 | 4 | AGAT | F: TCCACACGATAGACAGAGAGACAG |
|  |  |  | R: TTGAGAGACGCCGACCAC |
| Octoli_014 | 4 | ATAC | F: TGCATACACACACATACACA |
|  |  |  | R: TTGTTATTCCGATTCCTG |
| Octoli_015 | 4 | TAGA | F: TGCAATCACCTTAGGACCAAC |
|  |  |  | R: CAGCAATGGAGCTGAGAGTG |
| Octoli_016 | 4 | TATT | F: TGTGTATGTCTTAAGCCTCGT |
|  |  |  | R: TATAAGGAGTGGAGGAGCAA |
| ***Octoli_017** | 4 | TATG | F: AGCAACACGATGGCCTCTAC |
|  |  |  | R: AGTCCAACAAGCTTCGATCC |
| Octoli_018 | 4 | TTGC | F: CCAATAACTCGTGACATCTG |
|  |  |  | R: AACTACTTGTCCGATATACGC |
| Octoli_019 | 3 | CAC | F: CGCATGCGTTAAGTGACAG |
|  |  |  | R: CGTCCATAGACGTGAGGAAG |
| ***Octoli_020** | 3 | GTA | F: TTACTCATCTAGCCAATCAGTATGCAG |
|  |  |  | R: TAATACGGCCGTCGCTCTG |
| Octoli_021 | 3 | GTT | F: TTGCATTGGATGTGAATTATTA |
|  |  |  | R: TCTAAGGACTGAACGAGCTG |
| Octoli_022 | 3 | TGA | F: AGCCATGTGGTTGAGAACG |
|  |  |  | R: GCGTGCCTCTCTTCATCAG |
| ***Octoli_023** | 3 | GAT | F: GCCATGAATTCCAAGTAACTAACC |
|  |  |  | R: CATCGTCATACGCCATCATC |
| Octoli_024 | 3 | TAT | F: TCATGTTGAGAGTCGTTCCTG |
|  |  |  | R: TGGTGCTATTGAGTTAGACATGG |
| Octoli_025 | 3 | CTT | F: TTCTTCACCATCACCACAGG |
|  |  |  | R: AGCAGTACCAGCCAGATGC |
| Octoli_026 | 3 | GCG | F: GCGGTGGTGGTGGTAGTAG |
|  |  |  | R: TCATCAGCCTTGTTCTATGACTG |
| Octoli_027 | 3 | ATA | F: ACACCATCAAGCATGTCGTC |
|  |  |  | R: CACTTGTGTACTGGATCCGATG |
| Octoli_028 | 3 | TAT | F: GTATGAAGCAGAGGTGACGATG |
|  |  |  | R: CATGATTGCTGCTGACAAGG |
| Octoli_029 | 3 | TAG | F: CCAGCATCATCATCACCTTC |
|  |  |  | R: CTGTGTGCCTTGAGAGACG |
| Octoli_030 | 3 | ATC | F: CATTCCAGCGTGAAGCAAG |
|  |  |  | R: TTCTAACCACACAGCCATGC |
| Octoli_031 | 3 | ATC | F: TCTTAACATTCTGACTAACAATAATGG |
|  |  |  | R: GTCACTGGTGGATGATGATG |
| Octoli_032 | 3 | ATC | F: TTCACTGCACTTCCGAGT |
|  |  |  | R: CCACGTGTATGACTGGTACTT |
| Octoli_033 | 3 | TTC | F: TGATTGAATTCTTGTGGTATCT |
|  |  |  | R: AAGTACTATTGTATGGTGCTCAA |
| Octoli_034 | 3 | TGG | F: CATGTGTGCTTGTGTGGAAC |
|  |  |  | R: CCACCACGTATACTAACGAACG |
| Octoli_035 | 3 | TTA | F: TCAGTTAAGATAAGAAGCCATGACAG |
|  |  |  | R: GCCAGCTCACTGTGTTGTTG |
| Octoli_036 | 3 | GAT | F: GCTGCTGCTACTGATGATG |
|  |  |  | R: TCTATCATCATCCTCCTCCTC |
| Octoli_037 | 3 | GAT | F: TCCTGTTGAATGAATGTTAC |
|  |  |  | R: TGTTGATATCTCCTCATGTC |
| Octoli_038 | 3 | GTA | F: AACAATAATATGGCGGAACAG |
|  |  |  | R: ACTGACTGCTGCTGCTCTAC |
| Octoli_039 | 3 | ATG | F: AAGCATGGATGATGAAGAAT |
|  |  |  | R: ACGGACAACAGACAACAATA |
| Octoli_040 | 3 | GTG | F: CTGCATCTCTTCTGTGAAC |
|  |  |  | R: AGTTAGTAGTACGAAGAAGTAAGAA |
| Octoli_041 | 2 | AC | F: GTGATGGTGCAGAATGGAAG |
|  |  |  | R: ACCACGCACACACTATTGC |
| Octoli_042 | 2 | AC | F: CAGTGGTGATGGTGTGAG |
|  |  |  | R: GTTAGTAACCGACCACGAC |
| Octoli_043 | 2 | AC | F: AGATATGTATACCGTCGTTG |
|  |  |  | R: TTAATTAGTTAGTTAGTCGAGTAGTAG |
| Octoli_044 | 2 | TG | F: TTGCTTGTGTGTGGAGAAGG |
|  |  |  | R: CACACCTACCTACCACCTACCG |
| Octoli_045 | 2 | CA | F: GAGACTTACTTCGTTGTGTGG |
|  |  |  | R: AGGAGTGTGTCGTATATATAATAATTG |
| Octoli_046 | 2 | CA | F: GTTAGCCTTACACTCTGCTAATTCC |
|  |  |  | R: CGCGTCTGTATGTGTGTCG |
| Octoli_047 | 2 | TG | F: TTCCATTCTGAGGTGGAACAC |
|  |  |  | R: CGTTCGTCTCTCTCTTCTTCTCTC |
| Octoli_048 | 2 | AC | F: GCATATGTGTGCGTATAGTTG |
|  |  |  | R: GGTCGAAGGAAGTAATTAGTTATAG |

S7. Expected exclusion probabilities for the 5 microsatellite loci from S6 used in this study to evaluate multiple paternity in *Octopus oliveri*. Although exclusion probabilities for individual loci can be rather low, the combined power for all loci exceeds 95% even when neither parent is known.

| ﻿Expected exclusion probabilities | | | |
| --- | --- | --- | --- |
|  | Neither parent known | One parent known with certainty, one unknown | Parent pairs known |
| Octoli_3 | 0.552 | 0.714 | 0.881 |
| Octoli_7 | 0.468 | 0.643 | 0.826 |
| Octoli_17 | 0.138 | 0.257 | 0.393 |
| Octoli_22 | 0.665 | 0.799 | 0.936 |
| Octoli_23 | 0.530 | 0.695 | 0.866 |
| All Loci | 0.968 | 0.995 | 1.000 |

S8. Power analysis to determine the number of eggs needed to sample per clutch to detect multiple paternity in *Octopus oliveri*. We selected a sample size of 34 eggs, to have a 95% chance of detecting at least 10 possible sires.


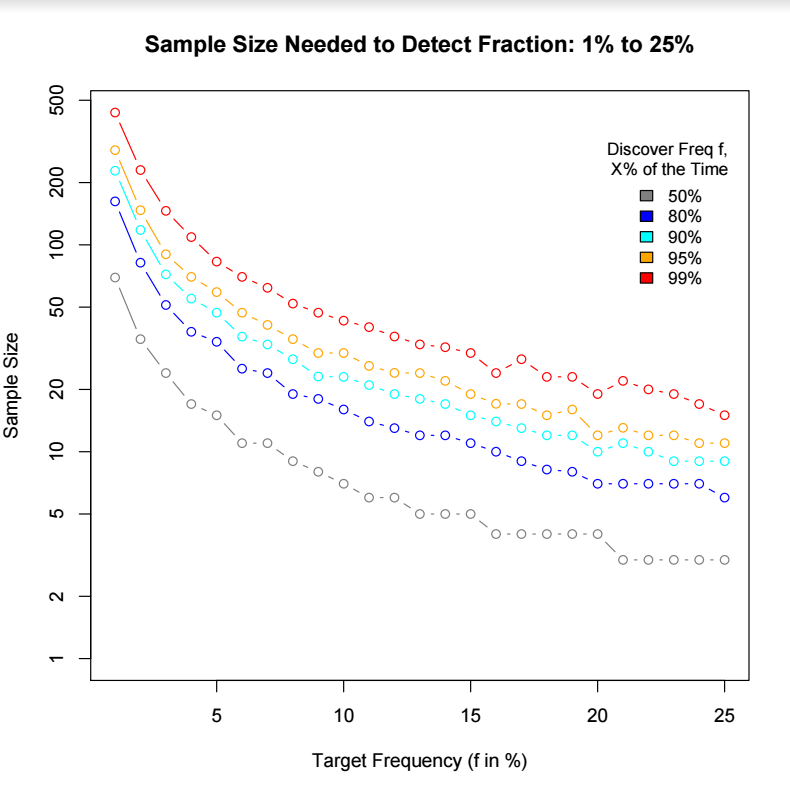


S9. Number of non-maternal alleles detected in a sample of 34 eggs for each of 8 experimental and 3 wild-caught females of *Octopus oliveri*. Data are presented for each locus and the minimum number of males needed to account for those alleles.

| Experimental Females | Locus Octoli_3 | Locus Octoli_7 | Locus Octoli_17 | Locus Octoli_22 | Locus Octoli_23 | Min no. of males (SLM) | Min no. of males (GERUD) |
| --- | --- | --- | --- | --- | --- | --- | --- |
| F1 | 6 | 4 | 2 | 7 | 5 | 4 | 5+ |
| F2 | 4 | 2 | 1 | 4 | 3 | 2 | 4 |
| F3 | 4 | 3 | 2 | 5 | 4 | 3 | 5 |
| F4 | 4 | 2 | 1 | 5 | 4 | 3 | 5 |
| F5 | 5 | 4 | 0 | 5 | 5 | 3 | 5* |
| F6 | 3 | 1 | 1 | 4 | 6 | 4 | 5+ |
| F7 | 5 | 1 | 1 | 6 | 5 | 3 | 5* |
| F8 | 4 | 2 | 3 | 4 | 4 | 3 | 5+ |
|  |  |  |  |  |  |  |  |
| Non-experimental females |  |  |  |  |  |  |  |
| F9 | 2 | 1 | 0 | 2 | 4 | 2 | 3 |
| F10 | 3 | 3 | 0 | 5 | 2 | 3 | 4 |
| F11 | 1 | 1 | 1 | 1 | 1 | 1 | 2 |

**+** indicates GERUD unable to calculate higher minimum number of sires due to program freezing,

*locus Octoli_17 removed from analysis and genotype array rerun to confirm result.

S10. Number of eggs per brood of experimental female *Octopus oliveri* sired by each male, as calculated by CERVUS, with 95% and 80% confidence levels. If none of the candidate fathers matched the offspring genotype, they were considered as sired by “other”.

| 95% Confidence | |  |  |  | 80% Confidence | |  |  |  |
| --- | --- | --- | --- | --- | --- | --- | --- | --- | --- |
| Female | 1st male | 2nd male | 3rd male | Other | 1st male | 2nd male | 3rd male | Other | N |
| Set 1 |  |  |  |  |  |  |  |  |  |
| F1 | 0 | 4 | 4 | 26 | 5 | 4 | 10 | 15 | 34 |
| F2 | -- | 11 | 0 | 23 | -- | 12 | 8 | 14 | 34 |
|  |  |  |  |  |  |  |  |  |  |
| Set 2 |  |  |  |  |  |  |  |  |  |
| F3 | -- | -- | 19 | 15 | -- | -- | 22 | 12 | 34 |
|  |  |  |  |  |  |  |  |  |  |
| Set 3 |  |  |  |  |  |  |  |  |  |
| F4 | 26 | ** | 1 | 7 | 28 | -- | 4 | 2 | 34 |
| F5 | 0 | ** | 16 | 18 | 2 | -- | 23 | 9 | 34 |
|  |  |  |  |  |  |  |  |  |  |
| Set 4 |  |  |  |  |  |  |  |  |  |
| F6 | 17 | 3 | 1 | 13 | 25 | 4 | 5 | 0 | 34 |
| F7 | 16 | 2 | 0 | 16 | 21 | 2 | 0 | 11 | 34 |
| F8 | 11 | 2 | 0 | 21 | 21 | 4 | 3 | 6 | 34 |
| Average | 11.67 | 3.14 | 5.13 | 17.38 | 17 | 5.2 | 9.38 | 8.63 | 34 |
| Total | 70 | 22 | 41 | 139 | 102 | 26 | 75 | 69 | 272 |

-- indicates no mating occurred, ** indicates paternal genotype missing


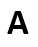

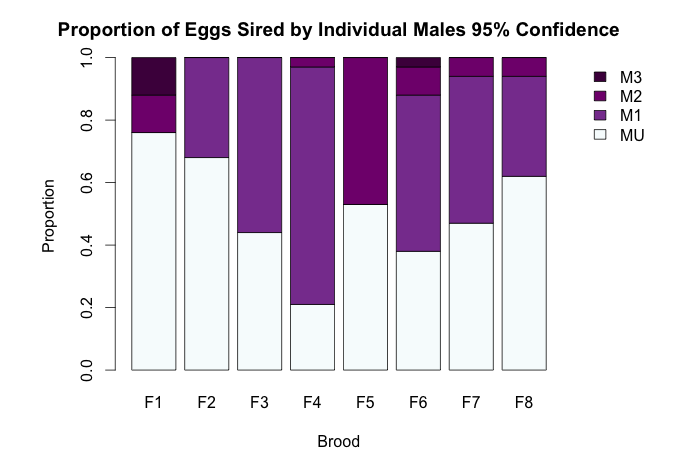


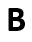

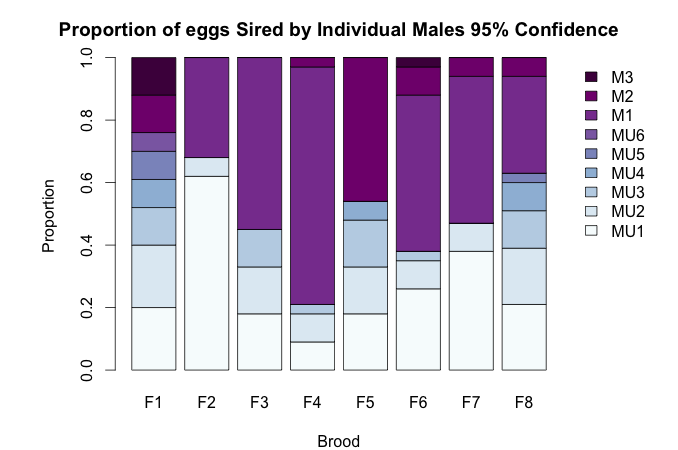


S11. A) Percentage of eggs sired by *Octopus oliveri* males ranked by order for each of the female broods. MU: Unknown males, M1: First male to mate in experimental trials, M2: Second experimental male, M3: Third male. B) Same data but with unknown males split into putative wild genotypes to show distribution among unknown mates.

S12. Number of unknown candidate male *Octopus oliveri* that contribute to each brood and number of eggs attributed to each male in mating trials.

| Female ID | Number of Unassigned Eggs | Number of Sires | Distribution of offspring among sires |
| --- | --- | --- | --- |
| F1 | 26 | 6 | 7, 7, 3, 2, 3, 4 |
| F2 | 23 | 2 | 21, 2 |
| F3 | 15 | 3 | 6, 5, 4 |
| F4 | 7 | 3 | 3, 3, 1 |
| F5 | 18 | 4 | 6, 5, 5, 2 |
| F6 | 13 | 3 | 9, 3, 1 |
| F7 | 16 | 2 | 13, 3 |
| F8 | 21 | 5 | 7, 6, 4, 3, 1 |
|  |  |  |  |
| Non-Experimental Females |  |  |  |
| F9 | 34 | 3 | 18, 11, 5 |
| F10 | 24 | 4 | 9, 9, 4, 2 |
| F11 | 34 | 2 | 30, 4 |

S13. AIC Model selection table of explanatory variables for the proportion of offspring sired by male *Octopus oliveri*, listed in order of the highest to lowest weighted. (Continued on next page).

| Explanatory variables | DF | AICc | Δ AIC | Weight |
| --- | --- | --- | --- | --- |
| male order x male size | 5 | 110.9 | 0 | 0.538 |
| male order x number of arch and pumps x male size | 6 | 114 | 3.06 | 0.116 |
| male order | 4 | 115.2 | 4.3 | 0.063 |
| male order x mating time x male size | 6 | 115.3 | 4.36 | 0.061 |
| male order x male size x removal of sperm packet | 6 | 115.3 | 4.38 | 0.057 |
| male size | 3 | 116.2 | 5.3 | 0.038 |
| male order x number of arch and pumps | 5 | 116.8 | 5.87 | 0.029 |
| male order x mating time | 5 | 117.5 | 6.62 | 0.02 |
| mating time | 3 | 117.8 | 6.92 | 0.017 |
| number of arch and pumps | 3 | 117.9 | 6.99 | 0.016 |
| number of arch and pumps x male size | 4 | 118 | 7.08 | 0.016 |
| removal of sperm packet | 3 | 118 | 7.12 | 0.015 |
| male order x mating time x number of arch and pumps x male size | 7 | 118.3 | 7.44 | 0.013 |
| male order x removal of sperm packet | 5 | 118.6 | 7.69 | 0.012 |
| male order x removal of sperm packet x number of arch and pumps x male size | 7 | 119 | 8.1 | 0.009 |
| male size x removal of sperm packet | 4 | 119.1 | 8.16 | 0.009 |

(Continued) AIC Model selection table; variables listed in order of the highest to lowest weighted.

| Explanatory variables | DF | AICc | Δ AIC | Weight |
| --- | --- | --- | --- | --- |
| male order x mating time x removal of sperm packet x male size | 7 | 120.4 | 9.51 | 0.005 |
| mating time x number of arch and pumps | 4 | 120.9 | 9.97 | 0.004 |
| number of arch and pumps x removal of sperm packet | 4 | 120.9 | 10.05 | 0.004 |
| mating time x removal of sperm packet | 4 | 121 | 10.14 | 0.003 |
| mating order x mating time x number of arch and pumps | 6 | 121.1 | 10.18 | 0.003 |
| male size x number of arch and pumps x removal of sperm packet | 5 | 121.7 | 10.84 | 0.002 |
| mating time x number of arch and pumps x male size | 5 | 121.7 | 10.84 | 0.002 |
| mating time x number of arch and pumps x removal of sperm packet | 5 | 121.7 | 10.84 | 0.002 |
| male order x mating time x removal of sperm packet | 6 | 121.8 | 10.93 | 0.002 |
| mating time x arch and pump x removal of sperm packet | 5 | 124.6 | 13.72 | 0.001 |
| mating time x male size x removal of sperm packet | 5 | 122.7 | 11.8 | 0.001 |
| male order x mating time x number of arch and pumps x male size x removal of sperm packet | 8 | 124.5 | 13.61 | 0.001 |
| mating time x number of arch and pumps x removal of sperm packet x male size | 6 | 126.1 | 15.22 | 0 |
| male order x mating time x number of arch and pumps x removal of sperm packet | 7 | 126.2 | 15.28 | 0 |
